# High-level expression of P21-Cdc/Rac-activated kinase 7 is closely related to metastatic potential and poor prognosis of colon carcinoma

**DOI:** 10.18632/oncotarget.10017

**Published:** 2016-06-14

**Authors:** Chao Li, Jian Chen, Yupeng Wang, Guohe Song, Chao Xiao, Dongwang Yan, Lin Zhong, Xing Sun, Xiaoliang Wang, Fudong Yu, Yang Yu, Huamei Tang, Zhihai Peng

**Affiliations:** ^1^ Department of General Surgery, Shanghai General Hospital, Shanghai Jiao Tong University School of Medicine, Shanghai 200080, People's Republic of China; ^2^ Department of Pathology, Shanghai General Hospital, Shanghai Jiao Tong University School of Medicine, Shanghai 200080, People's Republic of China

**Keywords:** PAK7, colon cancer, EMT, metastasis, prognosis

## Abstract

P21 protein (Cdc42/Rac)-activated kinase 7 (PAK7) can promote neurite outgrowth, induce microtubule stabilization, and activate cell survival signaling pathways. PAK7 expression was found to increase with colon carcinoma progression, but the prognostic value, clinical significance, and underlying mechanisms have not been explored. In my study, the expression of PAK7 was up-related at both the transcriptional and the translational levels in colon tumors compared to that in adjacent normal colon tissue. Patients with PAK7-positive tumors had a lower rate of overall survival (OS) and metastasis-free survival (MFS) (log-rank test, *P* < 0.001). A Cox proportional hazards model showed that PAK7 expression was an independent prognostic factor for OS (hazard ration [HR], 2.08; 95% confidence interval [CI], 1.16-3.73; *P* = 0.004) and MFS (HR, 2.88; 95% CI, 1.53-5.42; *P* < 0.001) in patients with colon cancer. Patients with tumors that were over-expressing PAK7 experienced metastasis, and died within a significantly shorter time after surgery (*P* < 0.001). Knockdown of PAK7 by a specific short hairpin RNA (shRNA) significantly suppressed the progression of epithelial to mesechymal transition (EMT), migration, and invasion of colon cancer cells *in vitro* and tumor growth *in vivo*. However, overexpression of PAK7 significantly promoted these processes. These findings indicate that aberrant PAK7 expression is associated with the occurrence of metastasis and poor clinical outcomes of human colon cancer by promoting the EMT, and the assessment of PAK7 expression might be helpful in predicting metastasis and prognostication for patients with colon cancer.

## INTRODUCTION

Colon cancer is responsible for more than half a million deaths worldwide each year [1]. About 90% of these deaths are caused by metastatic diseases [2]. Currently, the mainstay of treatment for colon cancer is surgical resection. The intent of surgical intervention may be either curative or palliative depending on the spread of disease and the possibility of complete tumor clearance via surgery [3]. Metastasis is the final step in solid tumor progression and is the main cause of failure of colon cancer therapy following surgery [4]. Metastasis is a multi-step process: invasion of tumor cells into the adjacent tissues, entry of tumor cells into the systemic circulation (intravasation), survival in circulation, extravasation to distant organs, and finally growth of cancer cells to produce secondary tumors [5].

The process through which tumor cells become metastatic is largely unknown. Metastatic cells are believed to be rare, and to evolve during late stages of tumor progression via a series of genetic changes. These genetic changes enable the cells to progress through the sequential steps that finally result in growth in distant organ microenvironments [6]. However, prognostic markers for metastasis are rare. Patients, including those with the same tumor stage, can progress to variable clinical outcomes [7]. Therefore, mechanistic understanding of colon cancer initiation and metastasis is an urgent need.

Epithelial-mesenchymal transition (EMT) is a cellular mechanism known to play a vital role in normal embryonic development [8]. EMT is also involved in tumor occurrence and progression. In particular, EMT plays an important role in promoting tumor invasion and metastasis [8]. One study found that EMT plays a key role in the migration and invasion of colon cancer [9]. Therefore, research on the occurrence of colon cancer EMT will help to reveal the mechanism that regulates the migration and invasion of colon cancer, and may provide a theoretical basis for the treatment of colon cancer.

PAK family members are known to be downstream effectors of Rac/Cdc42 GTPases, which have been implicated in the regulation of cytoskeletal dynamics, proliferation, and cell survival signaling [10–13]. The human *PAK7* gene is located on chromosome 20p12. The protein encoded by this gene is a member of the PAK family of Ser/Thr protein kinases. PAK7 is expressed primarily in the brain, and appears to be constitutively active [14–16]. It is capable of promoting neurite outgrowth, and thus, may play a role in neurite development [14–16]. PAK7 is an inhibitor of MARK2, a kinase that has been shown to induce microtubule disruption by phosphorylating microtubule-associated proteins such as tau [17]. A direct interaction between PAK7 and MARK2 leads to stable microtubules and dynamic actin [18]. The subcellular localization of this kinase is tightly regulated during cell cycle progression. In addition, PAK7 shuttles from the mitochondria to the nucleus, and the mitochondrial localization of PAK7, which leads to phosphorylation of Ser112 and Ser136 of BAD (a pro-apoptotic member of the Bcl-2 protein family), is vital to its effects on cell survival [19]. PAK7 can also activate Raf-1, an important effector of Ras-mediated signaling, and target it to the mitochondria, regulating its kinase activity and controlling Raf-1 dependent signaling in the mitochondria [20]. Apart from its anti-apoptotic properties, the role of PAK7 in gastric cancer tissues and cell lines has been demonstrated [21]. In a previous study, increased PAK7 expression was also found during colon carcinoma progression [22]. However, the prognostic value and the mechanism of PAK7 over-expression have not been analyzed.

In this study, we investigated the expression of PAK7 in human colon cancers, and analyzed its correlation with clinicopathologic features and survival. To our knowledge, this is, the first study to explore the relationship between the expression of PAK7 and EMT in colon cancer, and that further clarifies the role of PAK7 in the development of colon cancer. Thus, PAK7 is expected to be a potential therapeutic target for the treatment of colon cancer.

## RESULTS

### The overexpression of PAK7 in colon cancerous tissues

**Figure 1:**
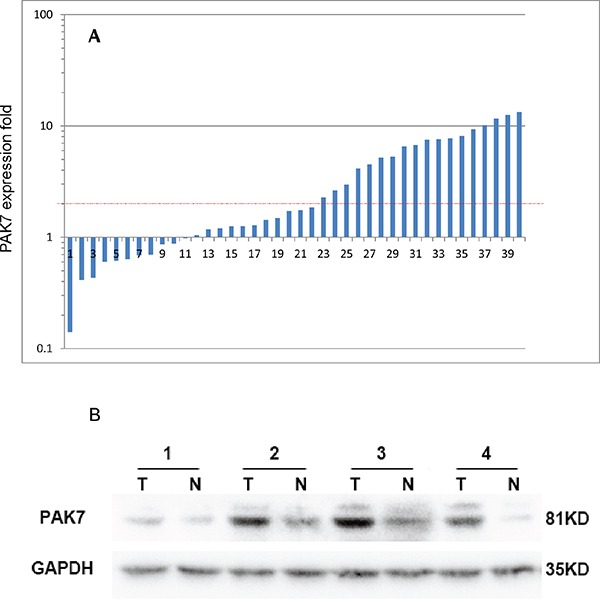
Expression of P21 protein (Cdc42/Rac)-activated kinase 7 (PAK7) in colon cancer tumors and adjacent normal mucosa **A.** Relative *PAK7* mRNA levels in 40 matched colorectal tumors compared with the levels in normal mucosa specimens. The logarithmic scale of 2-ΔΔCT is used to represent the fold change in quantitative real-time polymerase chain reaction detection; **B.** Western blotting analysis of PAK7 protein expression in four representative paired colon tumor/normal tissue pairings, glyceroldehyde-3-phosphate dehydrogenase (GAPDH) is used as the loading control.

Among the 40 paired cases for qPCR analysis, PAK7 mRNA expression was significantly higher in cancerous tissues than in adjacent normal mucosa (*P* < 0.001, Figure 1A). Subsequent western blotting confirmed that the PAK7 protein level was upregulated in cancerous tissues than in the adjacent normal tissues (Figure 1B), suggesting that PAK7 expression was elevated at both at the transcriptional and post-transcriptional levels.

### Expression of PAK7 and Ki67 correlated with clinicopathologic parameters

**Figure 2:**
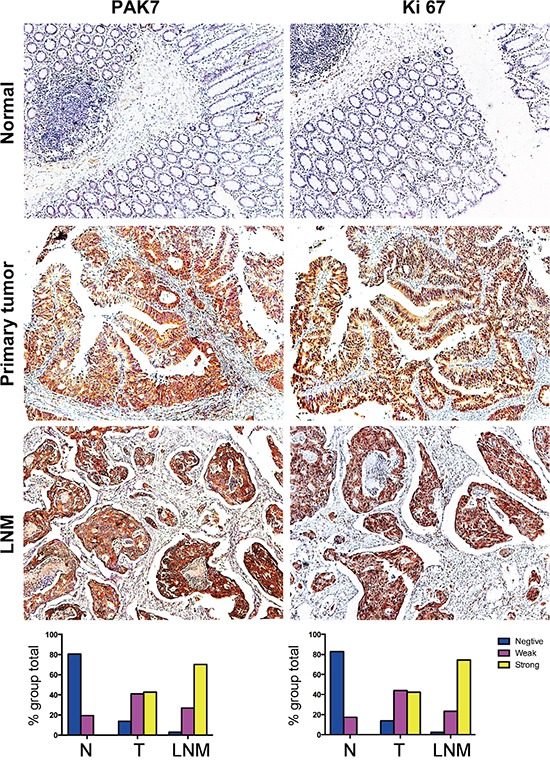
Representative photographs of PAK7 and Ki67 expression in normal colon, colon tumor, and nodal metastasis specimens Strong immunostaining of PAK7 and co-expression of Ki67 in primary colon cancer is noted. PAK7 and Ki67 overexpressed in lymph node metastasis (LNM). Original magnification 100×.

To determine the clinicopathologic significance of PAK7 expression, immunohistochemistry of a TMA containing 203 cases of primary colon cancer paired with noncancerous tissue and the available 66 cases of lymph node metastasis (LNM) was performed. 86 men and 117 women with a mean age of 65 ± 15 years (range, 22–95 years) were included. Of the 203 specimens on the paired TMA, 192 (94.6%) showed PAK7-negative staining in normal mucosa. In contrast, PAK7 expression was obvious in the majority of colon cancerous specimens, with strong, weak and negative in 48 (23.6%) cases, 74 (36.5%), and 81 (39.9%) cases, respectively (Table 1). Because LNM is a key step of the metastasis cascade in colon cancer, matched primary colon cancer and LNM tissues were evaluated. Of the 66 cases of LNM analyzed, 59 (89.4%) showed PAK7 overexpression (Table 1). Associations between the clinicopathologic factors and PAK7 expression are summarized in Table 2. Increased PAK7 expression was significantly correlated with the depth of tumor invasion (pT stage, *P* = 0.004), LNM (pN stage, *P* < 0.001), distant metastasis (M stage, *P* = 0.034), histologic grade (*P* < 0.001), and advanced AJCC cancer stage (*P* < 0.001) (Table 2). Factors not significantly associated with the staining included age, sex and tumor location. Overexpression of PAK7 was observed within LNM samples, and the levels of PAK7 expression in the nodal metastases were higher than those in the primary tumor and normal tissue (Figure 2). Moreover, we found that positive immunostaining for PAK7 was more frequently detected in specimens that stained positively for Ki67, suggesting a statistical correlation between PAK7 and Ki67 (*P* = 0.016).

**Table 1:** Immunohistochemical staining for PAK7 expression in normal colonic mucosa, cancerous tissue and lymph node metastases

Tissue sample	n	Expression of PAK7			*P* value
		Negative (%)	Weak (%)	Strong (%)	
Normal mucosa	203	192 (94.6)	11 (8.9)	0 (0)	<0.001*
Tumor	203	81 (39.9)	74 (36.5)	48 (23.6)	
LNM	66	7 (10.6)	13 (19.7)	46 (69.7)	

*P* value is based on Fisher's exact test, LNM: Lymph node metastasis.

**Table 2:** Association between clinicopathologic features and PAK7 protein expression

	n	PAK7 expression		*P* value
		Negative (n=81)	Positive (n=122)	
Age				
<65	81	28	53	0.206
≥65	122	53	69	
Gender				
Male	86	38	48	0.285
Female	117	43	74	
Location				
Right	84	32	52	0.988
Transverse	19	8	11	
left	20	8	12	
sigmoid	80	32	48	
pT stage				
pT1	8	3	5	0.004^*^
pT2	23	13	10	
pT3	76	39	37	
pT4	96	26	70	
pN stage				
pN0	108	66	52	<0.001^*^
pN1	61	22	39	
pN2	34	3	31	
M stage				
M0	184	66	118	0.034^*^
M1	19	2	17	
AJCC stage				
I	24	14	10	<0.001^*^
II	81	42	39	
III	80	25	55	
IV	18	0	18	
Vessel invasion				
No	189	79	110	0.043^*^
Yes	14	2	12	
Differentiation				
Well	99	58	41	<0.001^*^
Moderate	74	21	53	
poor	30	2	28	
Ki 67				0.016^*^
Negative	43	24	19	
Positive	160	57	103	

* Factors were compared by the Pearson χ^2^ text and *P* < 0.05 indicates a significant association between variables.

### High PAK7 expression is associated with poor clinical outcomes of colon cancer

**Figure 3:**
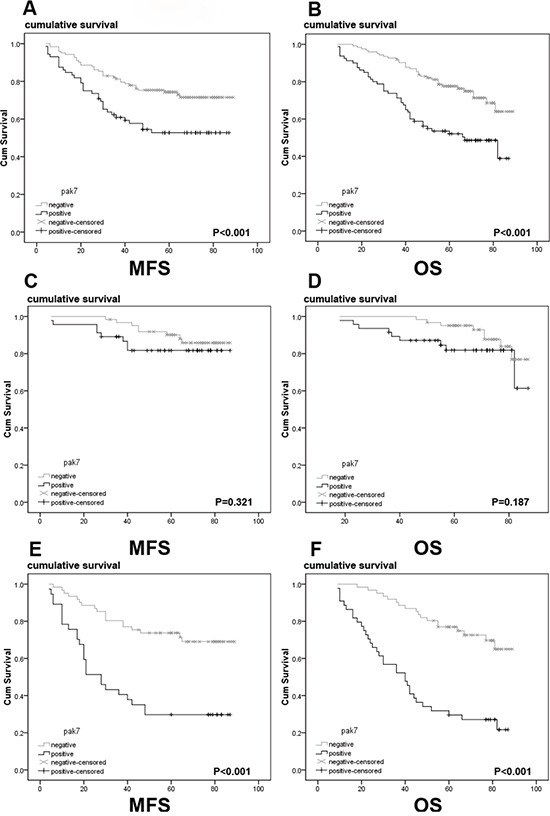
A Kaplan-Meier analysis with a log rank test of metastasis-free survival and overall survival in patients according to levels of PAK7 as determined by immunohistochemical staining In the group combining stage I, II, and III patients, both the **A.** MFS and **B.** OS rates of patients with PAK7-positive primary tumors is significantly lower than that of patients with PAK7-negative primary tumor (log-rank test, P < 0.001). In stage I and II patients, both **C.** MFS and **D.** OS rates of patients with PAK7-positive primary tumors do not significantly differ from that of patients with PAK7-negative primary tumors. However, in the group stage III patients, both **E.** MFS and **F.** OS rates of patients with PAK7-positive primary tumors are significantly lower than in patients with PAK7-negative primary tumors (log rank test, *P* < 0.001)

**Figure 4:**
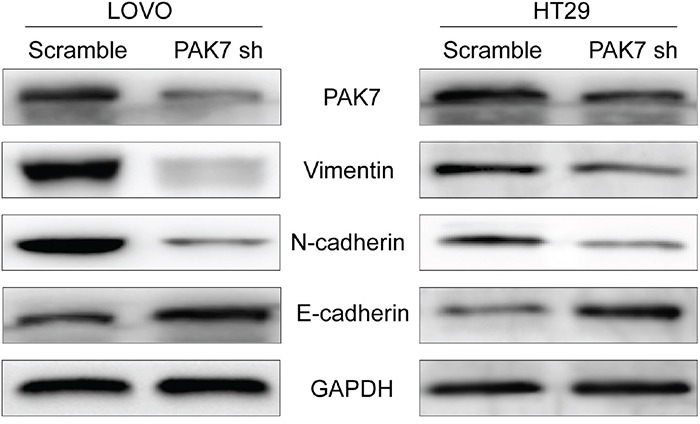
Western blot analysis of EMT markers Total protein lysates were prepared from LoVo and HT29 cells with PAK7 knockdown.

To evaluate the predictive significance of PAK7 for distant metastasis, we assessed PAK7 staining in 185 stage I-III patients who underwent radical colectomy, excluding 18 patients with advanced-stage disease who had undergone non-curative surgery to avoid the potential confounding influence of unresectable metastases. The proportion of samples with metastasis from primary colon cancer after curative surgery differed significantly between the PAK7-positive and PAK7-negative groups (Figure 3A). Patients with PAK7-positive tumors developed metastases earlier than those with PAK7-negative tumors (*P* < 0.001) (PAK7-positive, 38 of 104 patients [36.5%], mean follow-up 63.8 months [range 57.6–73.8 months]; PAK7-negative, 10 of 81 [12.3%], mean follow-up 83.2 months [76.8–87.3]). Positive PAK7 expression was associated with a nearly 3-fold increased risk of distant metastases (hazard ratio [HR], 3.80; [95% confidence interval 1.24-6.36]; *P* = 0.014). In the subgroup analysis, no significant difference was observed between stage I and II patients in the metastasis-free survival (MFS) rate in both PAK7-positive and -negative tumors (Figure 3C); however, in stage III patients, the MFS rate was significantly lower in patients with PAK7-positive tumors. Than in those who had PAK7-negative primary tumors (Figure 4E, log-rank test, *P* < 0.001). We used Kaplan–Meier analysis to show that the expression of PAK7 significantly correlated with the overall survival (OS) of colon cancer patients (log-rank test, *P* < 0.001, Figure 3B). Patients with PAK7-positive tumors had a significantly lower 5-year OS than those with PAK7-negative tumors (HR 2.08 [95% CI 1.16-3.73]). For stage I and II, no significant differences were observed in the OS between patients with PAK7-positive primary tumors and those with PAK7-negative primary tumors (Figure 3D). However, for stage III, patients with PAK7-positive tumors had a significantly lower rate of OS than those who had PAK7-negative primary tumors (Figure 3F, log-rank test, *P* < 0.001). In a multivariate analysis with clinicopathologic variables, such as clinical stage, differentiation grade, venous invasion, pN stage, and M stage, the expression of PAK7 was an independent prognostic marker to predict the patients' outcome (Table 3).

**Table 3:** Cox regression model analysis for overall survival (OS) and metastasis-free survival (MFS)

	OS		MFS	
	HR (95%CI)	*P* value	HR (95%CI)	*P* value
PAK7				
Positive VS. Negative	2.08 (1.16-3.73)	0.004*	2.88 (1.53-5.42)	<0.001*
Ki67				
High VS. low	1.17 (0.81-1.69)	0.413	1.31 (0.89-1.91)	0.166
Age				
≥65 VS. <65	1.27 (0.77-2.12)	0.349	1.26 (0.75-2.14)	0.384
Gender				
Female VS. Male	1.37 (0.82-2.77)	0.225	1.37 (0.80-2.35)	0.246
Location				
Transverse VS. Right	0.89 (0.36-2.19)	0.806	0.88 (0.33-2.38)	0.806
Left VS. Right	0.80 (0.33-1.91)	0.613	0.78 (0.32-1.90)	0.591
Sigmoid VS. Right	1.49 (0.88-2.51)	0.140	1.52 (0.87-2.65)	0.146
pT stage				
pT2 VS. pT1	1.13 (0.14-8.96)	0.908	0.95 (0.12-7.27)	0.961
pT3 VS. pT1	1.35 (0.29-1.35)	0.701	0.97 (0.10-4.72)	0.968
pT4 VS. pT1	3.30 (0.73-14.8)	0.120	2.28 (0.49-10.52)	0.290
pN stage				
pN1 VS. pN0	3.17 (1.62-6.21)	0.001*	2.47 (1.25-4.90)	<0.001*
pN2 VS. pN0	8.31 (3.99-17.31)	<0.001*	10.10 (4.82-21.20)	<0.001*
AJCC stage				
III + IV VS. I + II	2.64 (1.36-5.15)	0.006*	1.82 (1.15-4.45)	0.018*
M stage				
M1 VS. M0	5.29 (2.57-10.88)	<0.001*	3.80 (1.24-6.36)	0.014*
Vessel invasion				
Yes VS.No	2.75 (1.36-3.55)	0.043*	1.77 (1.35-2.70)	0.015*
Differentiation				
Moderate VS. Well	1.46 (1.21-2.00)	0.015*	2.60 (1.25-3.44)	0.025*
poor VS. Well	2.62 (1.32-3.20)	0.018*	2.80 (1.38-4.72)	0.021*

HR hazard ratio, CI confidence interval

### Altered PAK7 expression affects EMT induction in human colon cancer cells

**Figure 5:**
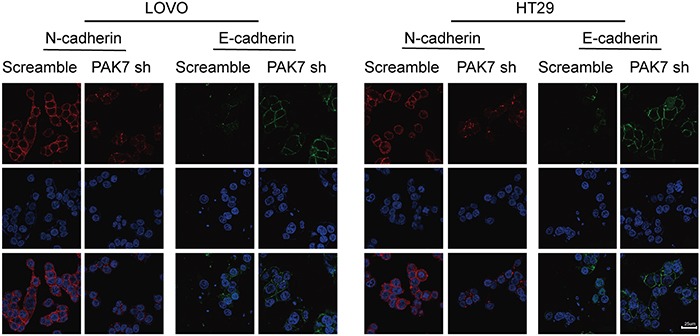
Cell immunofluorescence staining for E-cadherin (green), N-cadherin (red) and nuclei (DAPI; blue) LoVo and HT29 cells are transfected with control shRNA (“scramble”) or shRNA against PAK7 (“PAK7 sh”).

Because the overexpression of PAK7 examined by immunohistochemistry, was associated with colon cancer metastasis and clinical stage, (Table 2) we subsequently investigated the capability of PAK7 was to induce EMT in human colon cancer cells. Gene Set Enrichment Analysis (GSEA) identified significant association PAK7 and EMT (Supplementary Figure S1D). We chose cell lines expressing high levels of PAK7 (LoVo and HT29) as measured in previous experiments [8]. To determine the effect of altered PAK7 expression on EMT induction in colon cancer, we transfected PAK7-specific shRNA and the control vector into LoVo and HT29 cells. We found that down-regulated expression of PAK7 in LoVo and HT29 cells (Figure 4) significantly decreased the levels of vimentin and N-cadherin but increased E-cadherin expression in protein level (Figure 4). Immunofluorescence staining further revealed that E-cadherin (Figure 5) was strongly induced in PAK7 shRNA-transfected LoVo and HT29 cells, while the levels of N-cadherin were decreased in PAK7-silenced LoVo and HT29 cells (Figure 5). However, overexpression of PAK7 by gene transfer did the opposite (Supplementary Figure S1A, S1C). These results indicate that altered PAK7 expression affects EMT induction in colon cancer cells.

### Altered expression of PAK7 affected migratory and invasive ability of colon cancer cells *in vitro* and tumorigenecity *in vivo*

**Figure 6:**
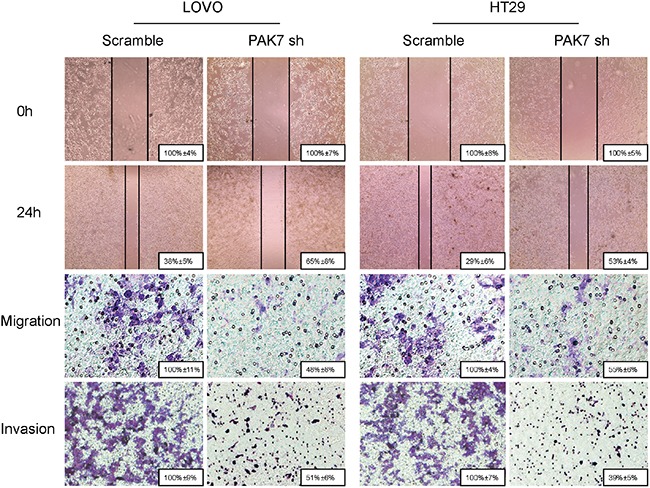
Influence of PAK7 in colon cancer cell migration and invasion LoVo and HT29 cells were transfected with PAK7 shRNA. For the cell scratch wound assay, the cultures were wounded by scratching and maintained at 37°C for 24 h. The cell cultures were then photographed. For the cell migration and invasion assay, the transfected cells were maintained at 37°C for 24 h. Representative images show migration of LoVo and HT29 cells via transwells without matrigel, measured by direct counting of trespassing cells. Representative images also show invasion of LOVO and HT29 cells through transwells with matrigel. Magnification, 200×.

To determine the effect of altered PAK7 expression on migration and invasion of colon cancer cells, the PAK7 transfected and PAK7 shRNA cells were wounded by scratching and maintained at 37°C for 24 h. The knockdown of PAK7 attenuated the flattening and spreading of LoVo and HT29 cells (Figure 6), whereas forced PAK7 expression strongly promoted the flattening and spreading of RKO cells (Supplementary Figure S1B).

**Figure 7:**
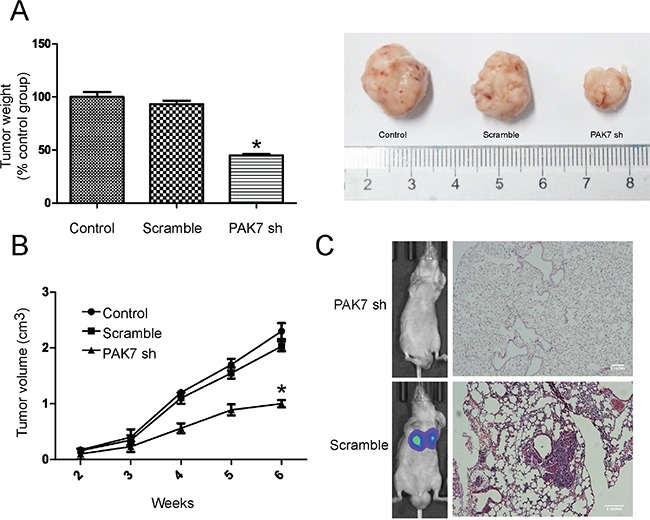
PAK7 inhibition suppresses proliferation and invasion of colon cancer cells *in vivo* **A.** Average weight of tumors harvested in the 6th week. **B.** Tumor volume curves of mice in different treatment groups. **C.** Representative images of luciferase signals of mice in 6 weeks after tail vein injection and representative hematoxylin and eosin-stained images of lung tissues. Values are the mean ± SEM from LoVo cells (the results using HT29 were the same).

Since PAK7 was correlated with migration and invasion in colon cancer cells, we further investigated the effect of PAK7 on the tumorigenic activity of colon cancer cell lines. Control cells (MOCK and shRNA) and PAK7 shRNA cells were subcutaneously injected into nude mice (n =5/group). The tumor growth curves and harvested tumor weights are shown in Figure 7A and 7B. The tumors formed from PAK7 shRNA cells grew slower than those formed from the control cells (Figure 7). After 4 weeks, the volume of tumors induced by the PAK7-suppressed cells was significantly reduced when compared to that induced by control cells (Figure 7).

Luciferase-tagged cells were injected into the tail vein of nude mice. After 6 weeks, bioluminescence imaging and H&E staining were performed (Figure 7C). The knockdown of PAK7 in LoVo and HT29 cells suppressed metastasis formation after tail-vein injection. Our data clearly indicate that PAK7 plays a critical role in the tumorigenicity of colon cancer and promotes invasion and metastasis of colon cancer.

## DISCUSSION

p21-activated kinases (PAK) are members of a family of Rho-associated serine/threonine kinases involved in a variety of cellular functions, including regulation of cell survival and cytoskeletal actin organization [25]. PAK7 possesses unique features, as exemplified by its mitochondrial localization and the requirement of this localization for its effects on cell survival. Since the knockdown of endogenous PAK7 in neuronal cell types renders these cells more sensitive to apoptosis, it is possible that this kinase plays a role in the survival of neurons under stress [18]. These findings imply that PAK7 may also play a role in carcinogenesis. In a prior study, it was found that PAK7 was overexpressed in a variety of colon cancer cells [8]. However, the prognostic significance of PAK7 in human colon cancer and its potential role in its pathogenesis and progression still remain unclear. Our study shows that (a) aberrant expression of PAK7 is significantly associated with more aggressive tumors, advanced clinical stage, LNM, and advanced tumor stage in colon cancer; (b) the PAK7-positive staining of colon cancer cells could be used to identify a highly increased risk of metastasis in patients after surgery, which might serve as a valuable prognostic maker (c) PAK7 promotes the migration and invasion of colon cancer by affecting ENT induction *in vitro* and *in vivo*.

In the fresh colon tissues examined in the current study, qPCR, western blotting and immunostaining showed that the elevated expression of PAK7 occurred at both transcriptional and post-transcriptional levels. In the present study, we specifically investigated the expression of PAK7 in TMA containing colon tumors and matched normal epithelium and LNM specimens. The strongest PAK7-positive staining was observed in the nodal metastasis specimens. We also found that PAK7 expression was significantly correlated with an advanced cancer biology, which was indicated by the invasion depth, LNM, and distant metastasis. These strong correlations suggest that PAK7 overexpression might promote tumor invasion and metastasis. PAK7 expression was associated with an increased risk of metastasis and was strongly linked to poor survival outcomes. On multivariate analysis, PAK7 appeared to be an independent prognostic factor for OS and MFS in colon cancer after surgical resection. Although the limitations include few numbers of patients with relatively short follow-up time, our results provide evidence that PAK7 might be a novel biomarker for predicting outcomes after colectomy in patients with colon cancer.

Consistent with the clinical assays, our experiments *in vivo* and *in vitro* support this theory. Altered PAK7 expression affected the ability of colon cancer cells migrate and invade. Invasion and metastasis are characteristic features of colon cancer and are the main factors related to the poor prognosis of patients with colon cancer [26]. In the present study, knockdown of PAK7 attenuated migratory and invasive abilities of colon cancer cells *in vitro* and tumorigenicity *in vivo*. However, the overexpression of PAK7 increases the migration and invasion of colon cancer cells *in vitro*. Our findings are consistent with other recent studies, which have identified an important role of PAK7 in tumorigenesis and metastasis of breast, pancreatic, and gastric cancer cells [21, 27, 28].

Although the understanding of metastatic processes has evolved greatly, mechanisms involved in colon cancer metastasis are not fully understood [29, 30]. Emerging evidence suggests that the EMT plays a crucial role in metastasis [31, 32]. In the current study, we found that expression of PAK7 is significantly associated with the EMT. Deregulated PAK7 expression could contribute to the progression of the EMT by up-regulating the epithelial cell marker E-cadherin and down-regulating the mesenchymal cell markers vimention and N-cadherin. The upstream and downstream of PAK7 are too complex. There are transfactors and non-coding RNA such as GATA1, miR-186, miR-129 [33–35]. Our next work in the future is to explore the special molecular mechanism and other pathways that may influence the expression and metastasis function of PAK7.

To summarize, this study provided critical insights into the role of PAK7 gene in the progression of colon cancer, and is the first to discover its relationship with EMT. Accumulation of PAK7 protein was significantly associated with poor prognosis in our study. PAK7 may be a therapeutic target for metastatic colon disease. Thus, patients with colon cancer with elevated expression of PAK7 might need more powerful adjuvant therapy and intensive follow-up. Future studies will address the applicability of this concept to other tissues.

## MATERIALS AND METHODS

### Patients and specimens

For tissue microarray (TMA) construction and immunohistochemical analysis, we used human colon tumor specimens obtained from 203 patients with colon cancer who underwent surgery at the General Surgery Department of Shanghai Jiao Tong University affiliated First People's Hospital between January 2001 and December 2003. Detailed information of these patients was described in a previous report [23]. Formalin-fixed, paraffin-embedded specimens were selected to represent all stages and histologic types of colon cancer. At least two pathologists confirmed the diagnoses, and staging was based on the basis of pathological findings according to the American Joint Committee on Cancer (AJCC). The patients' metastasis-free survival (MFS) and overall survival (OS) rate were defined as the interval from initial surgery to clinically and radiologically proven metastasis and death, respectively. The median patient follow-up time was 61 months after surgery (range, 9-89 months). The use of human specimens was approved by the Institutional Review Board of Shanghai Jiao Tong University Affiliated Shanghai First People's Hospital Medical Center.

### Cell culture and transient transfection

The LoVo, HT29 and RKO human colon cancer cell lines (Center of Shanghai Institutes for Biological Sciences, Type Culture Collection of the Chinese Academy of Sciences) were cultured at 37°C under high humidity, and 5% CO_2_ in Dulbecco's Modified Eagle's Medium(DMEM) (Gibco) supplemented with 10% fetal bovine strum (FBS) (Gibco), 1% streptomycin, and penicillin.

Three PAK7-siRNAs were designed and synthesized to get an effective sequence for gene knockdown studies. The most effective target sequence of the *PAK7* gene short interfering RNA (siRNA) were 5′-CGGGATTACCACCATGACAAT-3′. A scrambled siRNA (5′-TTCTCCGAACGTGTCACGT-3′) that had no homology with mammalian mRNA sequences was used as a negative control. LoVo and HT29 cells were transduced with 5 × 10^5^ transducing units/mL of lentivirus particles. The transduction efficiency was 90% and 86% for LoVo and HT29 cells, respectively. Human PAK7 was subcloned into the pcDNA3.1 (Invitrogen) in order to generate the pcDNA3.1-PAK7 plasmid. RKO cells were transfected with pcDNA3.1-PAK7 with the use of Lipofectamine 2000 Transfection Reagent (Invitrogen) according to the manufacturer's instructions. Antibiotic selection (1 μg/mL puromycin) was initiated for 7 days 24 h after transduction.

### Quantitative real-time polymerase chain reaction (qPCR)

Forty specimens of frozen tumor tissue and corresponding normal tissues were used for qPCR analysis. Total RNA was extracted according to the manufacturer's instructions (Qiagen, Hilden, Germany). First-strand cDNA was synthesized from 1 ug of total RNA according to the manufacturer's instructions (Promega Corporation, Madison, USA). qPCR analysis of the expression of PAK7 gene was performed using 2 ul of cDNA. Sequence of the forward primer was 5′-GGAAGCAACAGAGACGAGACT-3′, and that of the antisense primer was 5′-TTGTCAGGAGGATGGAGTCAC-3′. qPCR was performed on a Mastercycler ep realplex (Eppendorf, Hamburg, Germany) using the iQTM SYBR Green Supermix Kit (BIO-RAD, California, USA) according to the manufacturer's protocol. The cycling conditions were as follows: initial denaturation (1 min at 95°C) followed by 25 cycles of denaturation (1 min at 94°C), annealing (1 min at 94°C), and elongation (45 s at 72°C), with a final extension at 72°C for 5 minutes. Glyceraldehyde-3-phosphate dehydrogenase (GAPDH) was used as an internal control. The fold change 2-ΔΔCt in PAK7 expression in each paired sample was calculated using the formulas: PAK7ΔCt = (Avg.PAK7_Ct − Avg. GAPDH_Ct, RBBP ΔΔCt = PAK7ΔCt_tumor − PAK7ΔCt_non-tumor).

### Western blot analysis

Cellular protein was extracted from cells, frozen colon tumors, and adjacent normal tissue samples by using the radio immunoprecipitation assay lysis buffer and the concentration was determined using the BCA protein assay kit (Beyotime Biotechnology Co., Jiangsu, China). A standard western blot analysis of the lysates was performed with a primary monoclonal antibody to PAK7 (1:200, Abcam, UK) and an antibody to glyceraldehyde-3-phosphate dehydrogenase (GAPDH) (1: 1000, Abcam, UK) overnight at 4°C. After washing with Tris-buffered saline Tween-20 (TBST), the membranes were incubated with a secondary antibody against mouse immunoglobulin G. The membranes were washed, and protein was detected by enhanced chemiluminescence (Pierce Biotechnology, Rockford, IL, USA) according to the manufacturer's instructions. The abundance of PAK7, was normalized against the level of GAPDH expression.

### Imunohistochemistry

TMA slides were constructed by using the formalin-fixed, paraffin-embedded samples containing primary tumors and paired normal mucosa as previously described [23]. Among the 203 patients from the archives of the Department of Pathology of our hospital, 66 specimens had primary colon cancer paired with normal colonic mucosa and metastatic lymph nodes. Immunostaining was performed using a GT vision III kit (GK500705, Genetech, ShangHai, China). After antigen retrieval in citrate buffer (pH 6.0) for 10 minutes, the specimen slides were incubated overnight at 4°C with the primary antibody against PAK7 (1:100, Abcam, UK) and the proliferation marker Ki67 (1:100, Abcam, UK), and then incubated with the secondary antibody (GK500705, Genetech, Shanghai, China) for 30 minutes, at room temperature. Tissue sections were counterstained with Mayer's hematoxylin. Two researchers who were blinded to patient outcomes independently evaluated the intensity and extent of staining. Staining extension was scored as 0 (no staining), 1(mild staining), 2(moderate staining) or 3(intense staining). Staining area was scored as 0 (0%),1 (1%–25%), 2 (26%–50%), or 3 (>50%) according to the percentage of positive-stained cells [24]. The final staining score, which was the sum of the intensity and extension scores, was divided into three groups as follows: 0–2, negative expression; 3–4, weak expression; or 5–6, strong expression. In the case of any discrepant assessments, the slides were re-examined by both pathologists under a multi-head microscope until they reached consensus. Weakly and strong staining were considered as positive.

### Cell scratch-wound assay

For the cell scratch-wound assay, the colon cancer cells were grown in 6-well plates until confluent. A cell scratch-wound was generated by scraping with a 10-μL tip. After 24h, the wounded cells were photographed, and cell migration was identified by measuring gap sizes on multiple fields.

### *In vitro* migration and invasion assays

The migration and invasion assays were conducted in a modified 24-well Boyden chamber using an uncoated membrane and a membrane coated with Matrigel (BD Biosciences, San Jose, CA). Briefly, cells (5 × 10^4^) were seeded into the upper chambers and 10% FBS was filled in the lower wells. After 24h of incubation at 37°C in a CO_2_ incubator, the non-migratory and non-invasive cells in the upper chamber of the filter were removed with a cotton swab. The cells that migrated into the lower surface were fixed, stained and counted under a microscope in four randomly selected fields at 200× magnification.

### Cell immunofluorescence

LoVo, HT29 and RKO cells were cultured until 50-60% confluence before being fixed with 4% paraformaldehyde and being permeabilized with 0.3% Triton X-100. Cells were washed three times in phosphate-buffered saline (PBS) and incubated with PAK7, epithelial cadherin (E-cadherin) and neural cadherin (N-cadherin) (Abcam, UK) primary antibodies at 4°C overnight then, incubated with corresponding Alexa Fluor-conjugated secondary antibodies (Life Technologies, USA) at room temperature for 1 h, and mounted using ProLong® Gold Antifade Reagent with DAPI (Life Technologies, USA). Microscopic images of the cells were obtained using a Leica inverted fluorescence microscope (Leica Microsystems, Wetzlar, Germany) with ProgRes Image Capture Software (JENOPTIK Optical Systems, Jena, Germany) and a Leica Confocal LAS-AF SP5 System (Leica Microsystems, Germany).

### Tumorigenicity assay

LoVo and HT29 cancer cells with stably silenced PAK7 (sh-PAK7) or control cells (sh-Control and mock) (1×10^6^ cells; n=5 per group) were subcutaneously injected into six-week-old athymic nude mice. Tumor volume and body weight were measured once a week and the mice were sacrificed after 6 weeks. The cells (silenced PAK7 or control) were labeled with firefly luciferase and injected into the lateral tail vein of nude mice. Six weeks after the tail vein injection, bioluminescence imaging was performed on an IVIS Illumina System (Caliper Life Sciences, Hopkinton, MA) and the mice were sacrificed and examined for lung metastases by hematoxylin and eosin (H & E) staining. All procedures involving mice were conducted in accordance with Shanghai Jiao Tong University affliated Shanghai First People's Hospital Animal Care guidelines. All efforts were made to minimize animal suffering, to reduce the number of animals used, and to utilize possible alternatives to *in vivo* techniques.

### Statistical analysis

The 2-tailed χ^2^ test and Fisher's exact test were used to determine the statistical significance of differences between experimental groups. The survival rates were calculated by using the Kaplan-Meier method. A log-rank test was used to compare the survival curves. A Cox proportional hazards model was used to calculate multivariate hazard ratios for the variables. A *P* value of less than 0.05 was considered to be statistically significant. All statistical analyses were carried out with the SPSS 21.0 statistical software package (SPSS Inc., Chicago, IL).

## SUPPLEMENTARY FIGURE


