# Correction: High-level expression of P21-Cdc/Rac-activated kinase 7 is closely related to metastatic potential and poor prognosis of colon carcinoma

**DOI:** 10.18632/oncotarget.18923

**Published:** 2017-07-03

**Authors:** Chao Li, Jian Chen, Yupeng Wang, Guohe Song, Chao Xiao, Dongwang Yan, Lin Zhong, Xing Sun, Xiaoliang Wang, Fudong Yu, Yang Yu, Huamei Tang, Zhihai Peng

**Present**: Two images of the same scene with different brightness were accidentally included in Figure 6.

**Correct**: The proper Figure 6 is shown below.

Original article: Oncotarget. 2016; 7:46042-46055. https://doi.org/10.18632/oncotarget.10017

**Figure 6:**
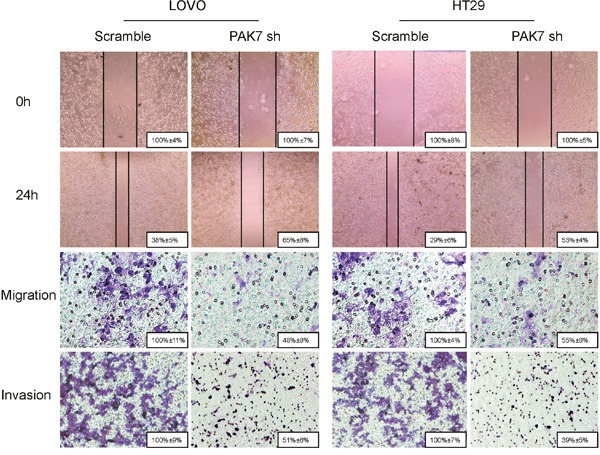
Influence of PAK7 in colon cancer cell migration and invasion LoVo and HT29 cells were transfected with PAK7 shRNA. For the cell scratch wound assay, the cultures were wounded by scratching and maintained at 37 °C for 24 h. The cell cultures were then photographed. For the cell migration and invasion assay, the transfected cells were maintained at 37°C for 24 h. Representative images show migration of LoVo and HT29 cells via transwells without matrigel, measured by direct counting of trespassing cells. Representative images also show invasion of LOVO and HT29 cells through transwells with matrigel. Magnification, 200×.

