# Supplementary material for: High-level expression of P21-Cdc/Rac-activated kinase 7 is closely related to metastatic potential and poor prognosis of colon carcinoma
Source: Oncotarget. 2016 Jun 14;7(29):46042–55. doi: 10.18632/oncotarget.10017 (PMC5216780; doi:10.18632/oncotarget.10017)
Supplement: Supplementary file 1 [file oncotarget-07-46042-s001.pdf]

High-level expression of P21-Cdc/Rac-activated kinase 7 is closely related to metastatic potential and poor prognosis of colon carcinoma

SUPPLEMENTARY FIGURE

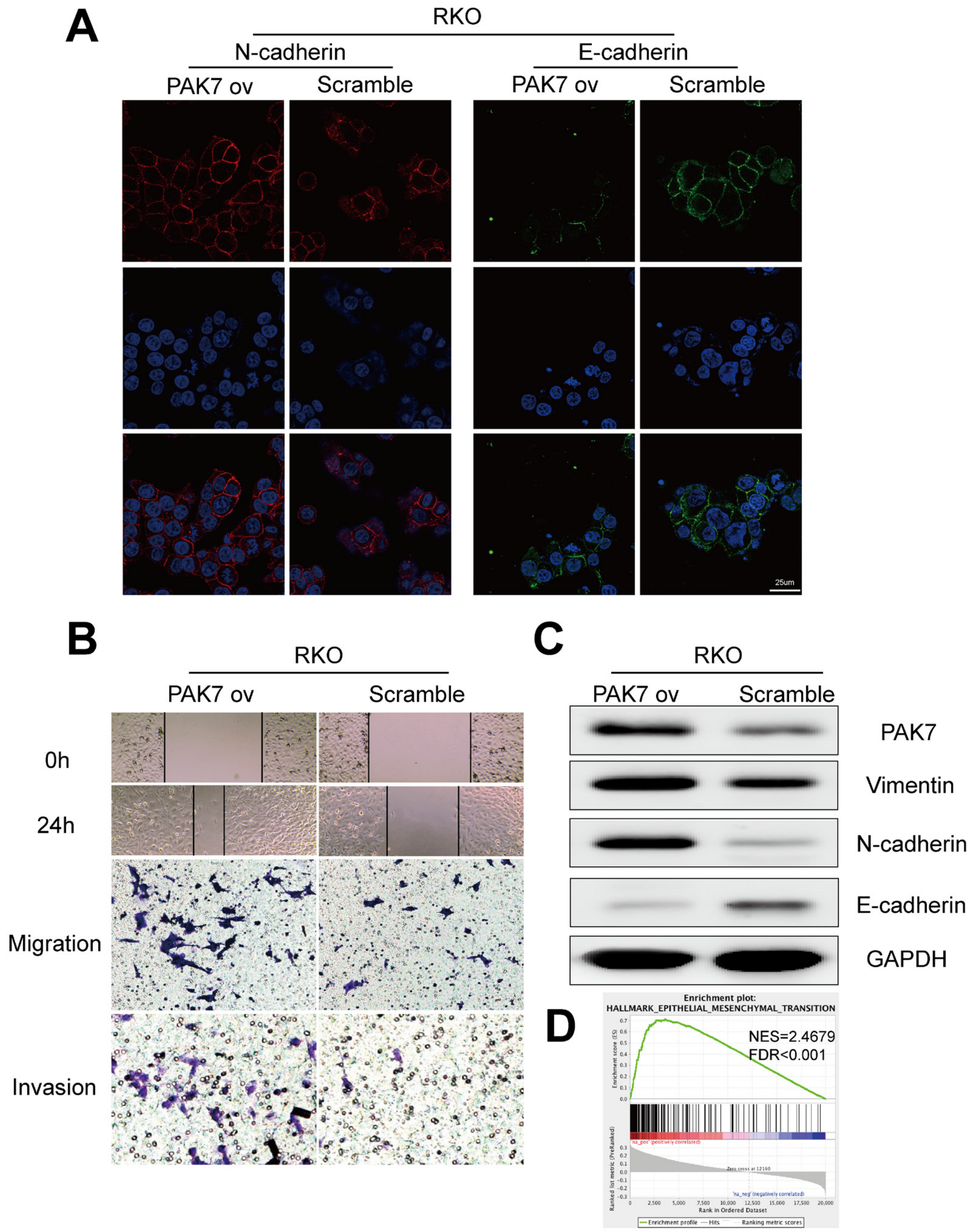

Supplymentary Figure S1: Influence of PAK7 in PAK7-overexpressed RKO cells. A. Cell immunofluorescence staining for E-cadherin (green), N-cadherin (red) and nuclei (DAPI; blue). The overexpression of PAK7 significantly promoted the occurrence of EMT. **B.** The overexpression of PAK7 promoted the migration and invasion of colon cancer cells. **C.** Western blot analysis showed that E-cadherin was decreased in PAK7-overexpressioned RKO cells, while the levels of N-cadherin were increased. **D.** Gene Set Enrichment Analysis (GSEA) identified significant association PAK7 and EMT.
